# Papillary Cystadenoma: An Incidental Finding in Tubal Ligation

**DOI:** 10.1155/2018/7964238

**Published:** 2018-04-23

**Authors:** Tabitha Lynn Ward, Neda Zarrin-Khameh

**Affiliations:** Department of Pathology, Baylor College of Medicine, One Baylor Plaza, Houston, TX 77030, USA

## Abstract

von Hippel-Lindau disease (vHLD) is a rare autosomal dominant disorder with multiple benign and malignant tumors of different organs. We report a papillary cystadenoma of the mesosalpinx found in close association with an adenomatoid tumor discovered incidentally following tubal ligation in a patient with vHLD.

## 1. Introduction

von Hippel-Lindau disease (vHLD) is a rare autosomal dominant disorder and can present by benign and malignant tumors of the eyes, central nervous system, kidneys, adrenals, and pancreas. Most tumors develop between the ages of 20 and 40 years [1]. A rare but well documented lesion in this disease is papillary cystadenoma of the epididymis which is seen in about 10% of men with vHLD [2]. The analogous female counterpart to this lesion was first described in the broad ligament by Gersell and King in 1988. It was used to be called adnexal papillary tumor of probable mesonephric origin.

## 2. Case Presentation

The patient is a 37-year-old woman (gravida 5, para 2) with type 1 von Hippel-Lindau disease and a history of hemangioblastomas in the cerebellum, frontal lobe, spine, retinas, and kidneys. She had a previous diagnosis of renal cell carcinoma status after radioablation at the age of 24. She presented at 34 weeks and 3 days of gestation with limited prenatal care and hypertension concerning for preeclampsia with severe features. She underwent an uncomplicated cesarean section delivery with bilateral tubal ligation. The placenta and fallopian tubes were sent to pathology for evaluation.

**Figure 1:**
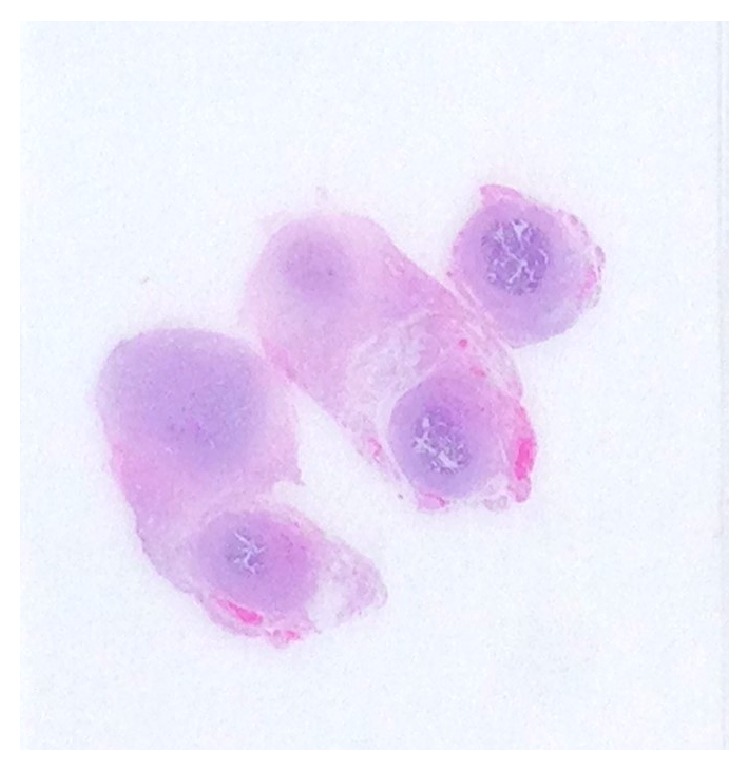
H&E stained cross section of left fallopian tube and adjacent nodule.

The submitted segment of left fallopian tube had a 0.9 × 0.7 × 0.5 cm tan-white nodule abutting the lumen of the fallopian tube (Figure 1). The cut surface of the nodule was tan-white and smooth. The right fallopian tube segment was unremarkable. The placenta had a 0.6 cm in the largest dimension chorangioma but otherwise was unremarkable.

**Figure 2:**
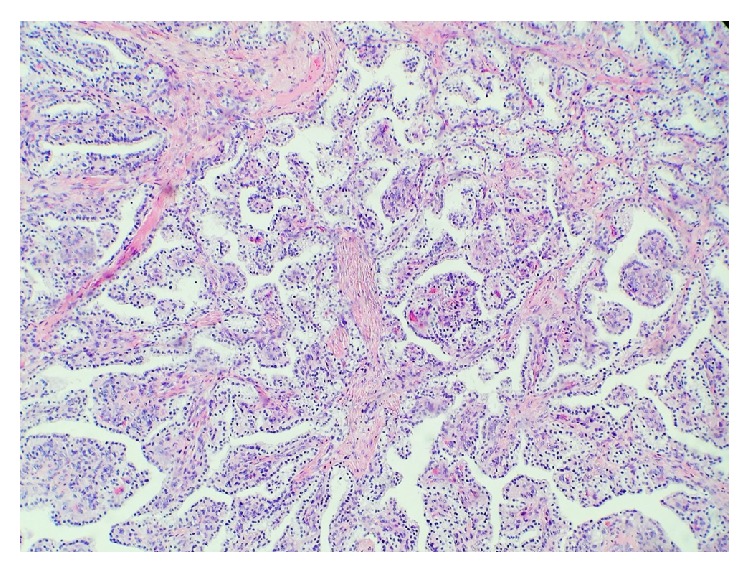
Papillary portion of nodule adjacent to left fallopian tube.

**Figure 3:**
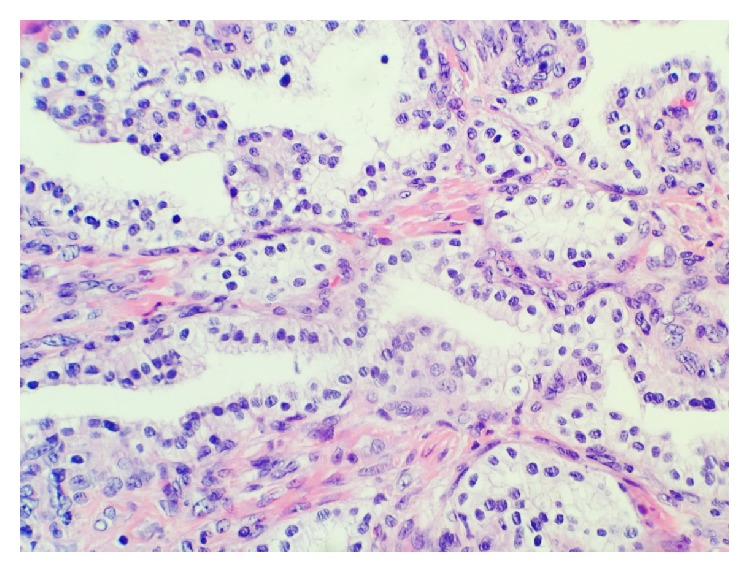
Papillae lined by clear cuboidal to columnar epithelium with bland appearing nuclei.

**Figure 4:**
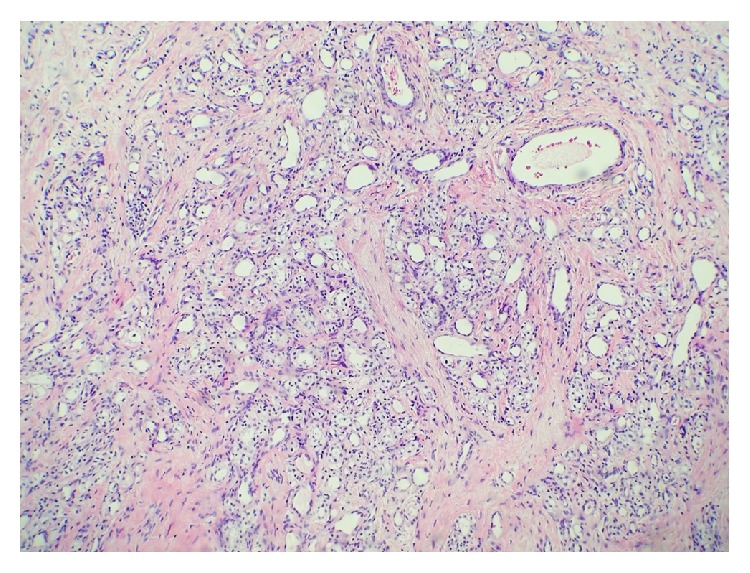
Lesion composed of small cystic spaces adjacent to papillary lesion.

**Figure 5:**
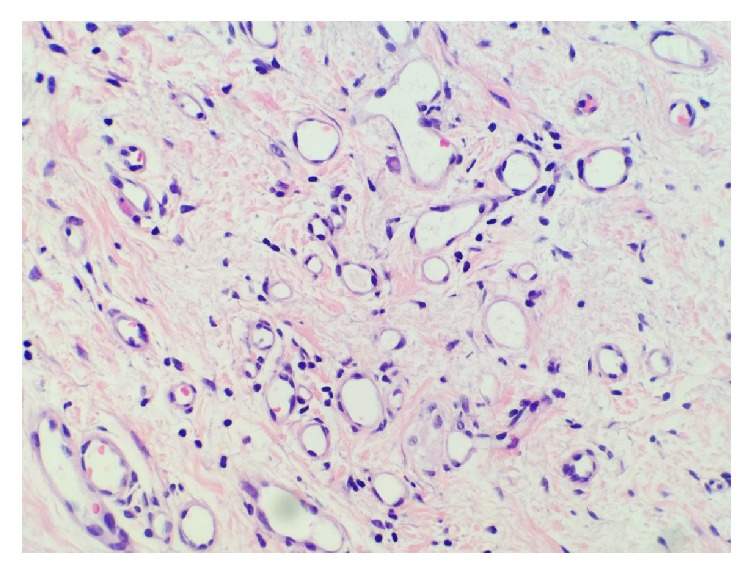
Cystic spaces lined by bland flattened epithelial cells with surrounding fibromuscular stroma.

Microscopic examination of the left fallopian tube nodule showed two lesions. The larger lesion had a papillary architecture lined by a single layer of nonciliated cuboidal to columnar cells with clear cytoplasm and bland nuclei (Figures 2 and 3). Adjacent to the papillary lesion was a lesion composed of small cystic spaces lined by flattened epithelium with bland nuclei and surrounded by loose fibromuscular stroma (Figures 4 and 5). No mitoses, necrosis, or atypical cells were seen in either lesion.

Periodic acid Schiff (PAS) staining of the papillary lesion highlighted the cytoplasmic contents of the clear cells. PASD was negative, indicating cytoplasmic glycogen. Tumor cells were positive for calretinin, D2-40, EMA, and WT1 (rare cells) and negative for PR, RCC, PAX8, and PAX5. Cytomorphology and staining patterns of the lesions were consistent with a papillary cystadenoma and an adjacent adenomatoid tumor.

## 3. Discussion

Although papillary cystadenomas of the epididymis have been well documented, only a limited number of cases of papillary cystadenomas of the mesosalpinx have been reported in women with vHLD. A single case reported that the investigations excluded VHLD indicating that these neoplasms may not invariably be associated with this disease in women [3].

Almost all reported lesions have been benign with most occurring bilaterally. Low-grade malignant behavior with peritoneal metastases was reported in a patient with symptoms of vHLD and LOH markers. Even in that patient no other metastases were found in a 15-year follow-up [4].

Papillary cystadenomas are usually reported as grossly or radiologically cystic lesions; however, our case was found incidentally as a 0.8 cm firm nodule. They are usually <0.5 cm.

It has a complex papillary pattern with short and blunted papillae. The stroma of the papillae varies in cellularity and may be hyalinized and fibrous. The papillae are usually lined by a single cell layer of low-cuboidal, nonciliated cells with clear or eosinophilic cytoplasm and bland nuclei. Mitotic figures are usually not seen. The cores project into a cystic space with eosinophilic material. Solid and tubular patterns can also be seen.

Papillary cystadenoma bears a striking histologic resemblance to recently described clear cell papillary renal carcinoma and should not be mistaken for its malignant counterpart clear cell renal carcinoma especially in vHLD patients that may already carry a diagnosis of renal cell carcinoma like our patient. If there is a suspicion for metastasis, the RCC antigen can be used to rule out metastatic renal cell carcinoma [5]. Papillary cystadenoma is diffusely CK7 positive. One study reported strong positivity with CD10 in the mesosalpinx of a woman with vHLD, while the 4 epididymal cases were negative [6]. Loss of heterozygosity of vHL gene with at least one polymorphic marker was reported in all of the 5 studied cases [7]. Other differential diagnoses include clear cell papillary cystadenoma, cystadenofibroma of Mullerian type with prominent papillary architecture. The latter has much larger and less complex architecture with ciliated cells.

One of the differential diagnoses is serous cystadenomas which are occasionally encountered in the mesosalpinx. They are frequently unilocular and, like their counterparts in the ovary, typically exhibit a dense, collagenized wall and an epithelial lining that varies from essentially flat to focally complex. Hydrosalpinx and hydatid of Morgagni are also considered as a differential diagnosis (Kayaalp, Heller & Mejudar, 2000).

Low-grade malignant behavior with peritoneal metastases was reported in a patient with symptoms of vHLD and LOH markers. Even in that patient no other metastases were found in a 15-year follow-up [4].
